# Life-long spontaneous exercise does not prolong lifespan but improves health span in mice

**DOI:** 10.1186/2046-2395-2-14

**Published:** 2013-09-16

**Authors:** Rebeca Garcia-Valles, Mari Carmen Gomez-Cabrera, Leocadio Rodriguez-Mañas, Francisco J Garcia-Garcia, Ana Diaz, Inma Noguera, Gloria Olaso-Gonzalez, Jose Viña

**Affiliations:** 1 Department of Physiology, Faculty of Medicine, University of Valencia, Fundacion Investigacion Hospital Clinico Universitario/INCLIVA, Av. Blasco Ibañez, 15, Valencia 46010, Spain; 2 Red Temática de Investigación Cooperativa en Envejecimiento y Fragilidad (RETICEF), Instituto de Salud Carlos III, Servicio de Geriatría, Hospital Universitario de Getafe, Ministerio de Sanidad y Consumo, Madrid, Spain; 3 Virgen del Valle Geriatric Hospital, Toledo, Spain; 4 UCIM, University of Valencia, Valencia, Spain

**Keywords:** Longevity, Sarcopenia, BDNF, Successful aging, Mitochondria, Frailty

## Abstract

**Background:**

Life expectancy at birth in the first world has increased from 35 years at the beginning of the 20th century to more than 80 years now. The increase in life expectancy has resulted in an increase in age-related diseases and larger numbers of frail and dependent people. The aim of our study was to determine whether life-long spontaneous aerobic exercise affects lifespan and healthspan in mice.

**Results:**

Male C57Bl/6J mice, individually caged, were randomly assigned to one of two groups: sedentary (n = 72) or spontaneous wheel-runners (n = 72). We evaluated longevity and several health parameters including grip strength, motor coordination, exercise capacity (VO_2max_) and skeletal muscle mitochondrial biogenesis. We also measured the cortical levels of the brain-derived neurotrophic factor (BDNF), a neurotrophin associated with brain plasticity. In addition, we measured systemic oxidative stress (malondialdehyde and protein carbonyl plasma levels) and the expression and activity of two genes involved in antioxidant defense in the liver (that is, glutathione peroxidase (GPx) and manganese superoxide dismutase (Mn-SOD)). Genes that encode antioxidant enzymes are considered longevity genes because their over-expression may modulate lifespan. Aging was associated with an increase in oxidative stress biomarkers and in the activity of the antioxidant enzymes, GPx and Mn-SOD, in the liver in mice. Life-long spontaneous exercise did not prolong longevity but prevented several signs of frailty (that is, decrease in strength, endurance and motor coordination). This improvement was accompanied by a significant increase in the mitochondrial biogenesis in skeletal muscle and in the cortical BDNF levels.

**Conclusion:**

Life-long spontaneous exercise does not prolong lifespan but improves healthspan in mice. Exercise is an intervention that delays age-associated frailty, enhances function and can be translated into the clinic.

## Background

World life expectancy has more than doubled over the past two centuries [1]. Life expectancy at birth in the first world has increased from 35 years at the beginning of the 20th century to more than 80 years now. Development of preventive medicine, improvements in nutrition and the use of antibiotics have probably been the main factors responsible for this important change which has been more pronounced in the last 100 years than in the previous 2,000. This transformation in the duration of life means that the number of older people has skyrocketed [2]. In developed countries, people over 65 years of age will represent 35% of the population by 2050. A concern of health providers is whether increasing longevity will increase disability, thus imposing an increasing financial burden [3]. Rather than extending lifespan we should be interested in an increased healthspan, the portion of the life span during which function is sufficient to maintain autonomy, control, independence, productivity and well-being [4]. Maximizing healthspan and preventing dysfunction are at least as important as extending lifespan [4,5].

Limits to healthspan include disability, frailty, chronic diseases and, of course, lifespan [6]. Frailty is a geriatric syndrome, defined by the presence of three or more of the following criteria: unintentional weight loss, self-reported exhaustion, weakness, slow walking speed and low physical activity [7]. Thus, the maintenance of the neuromuscular function is critical in the prevention of frailty [8]. Interest in this syndrome has been growing over the last decade because frailty is the main risk factor for disability in older people and it also forewarns of other adverse outcomes, such as falls, hospitalization and death [7,9]. In fact, the European Union has recently launched an effort to reach a consensual clinical definition of frailty (FOD-CC. Health.2010.2.2.2-5). Frailty results from age-related cumulative declines across multiple physiological systems, leading to impaired homeostatic reserve and a reduced capacity of the organism to withstand stress, thus increasing vulnerability to adverse health outcomes. Physical exercise is a very promising intervention for the modulation of both healthspan and lifespan in a number of species [10-12]. The benefits of regular exercise go beyond longevity [10]. Life-long physical exercise has become one of the key strategies in the prevention and treatment of chronic, degenerative diseases among older people. In animals, physical activity by means of spontaneous wheel-running confers cardiovascular, metabolic and psychological benefits [13,14].

Thus, the primary aim of our study was to develop an intervention (that is, spontaneous exercise) that could increase survival but that could also enhance function, delay frailty and be easily translated into the clinic. Due to the current lack of a test for frailty in rodents we performed four different physiological measurements: grip strength, motor coordination, exercise capacity and skeletal muscle mitochondrial biogenesis, which have been linked to clinically relevant age-related frailty. We also evaluated brain-derived neurotrophic factor (BDNF) as an indicator of brain plasticity, in addition to oxidative stress markers (malondialdehyde and carbonylated protein plasma levels) and the expression and activity of two genes involved in the antioxidant defense (that is, glutathione peroxidase (GPx) and manganese superoxide dismutase (Mn-SOD).

## Results

### Longevity curve and running wheel activity

**Figure 1:**
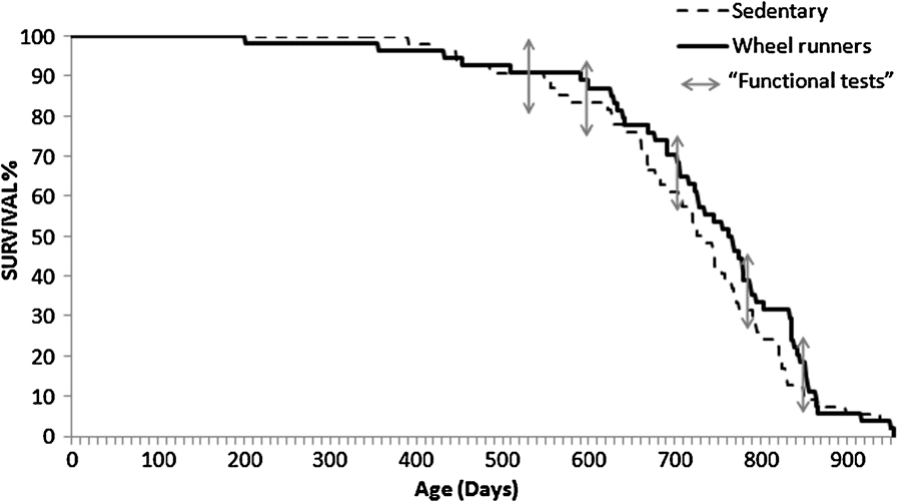
**Survival curves of cohorts of sedentary (n = 72) and spontaneous wheel-running mice (n = 72).** The Kaplan-Meier representation of the two groups is shown. The median lifespan of the sedentary group was 750 days and it was 770 days in the wheel-runners. Maximal lifespan in both groups was 950 days. The functional tests were performed at different survival time points (3, 17, 20, 23, 26, and 29 months old) as shown by arrows in the longevity curve.

Figure 1 shows the effect of lifelong spontaneous exercise on longevity in mice. Exercise does not cause an increase in either average lifespan or maximal lifespan. Maximal lifespan was defined as the age at which the longer-lived animal died. In our mice it was 950 days. Average lifespan was defined as the age at which 50% of the animals died. It was 750 days for sedentary mice and 770 for wheel-runners (*P* = 0.09). Our mice ran an average of 4.6 ± 1.5 km.d^-1^ at the beginning of the experiment. As in previous studies, there was a progressive decline in the distance run by the mice with advancing age [10,15]. Seventeen month-old mice ran approximately 0.5 km.d^-1^ and, as expected, this distance declined steadily during the life of the animals to the point that the very old ones (29 months and older) ran less than 0.1 km.d^-1^.

### Grip strength

**Figure 2:**
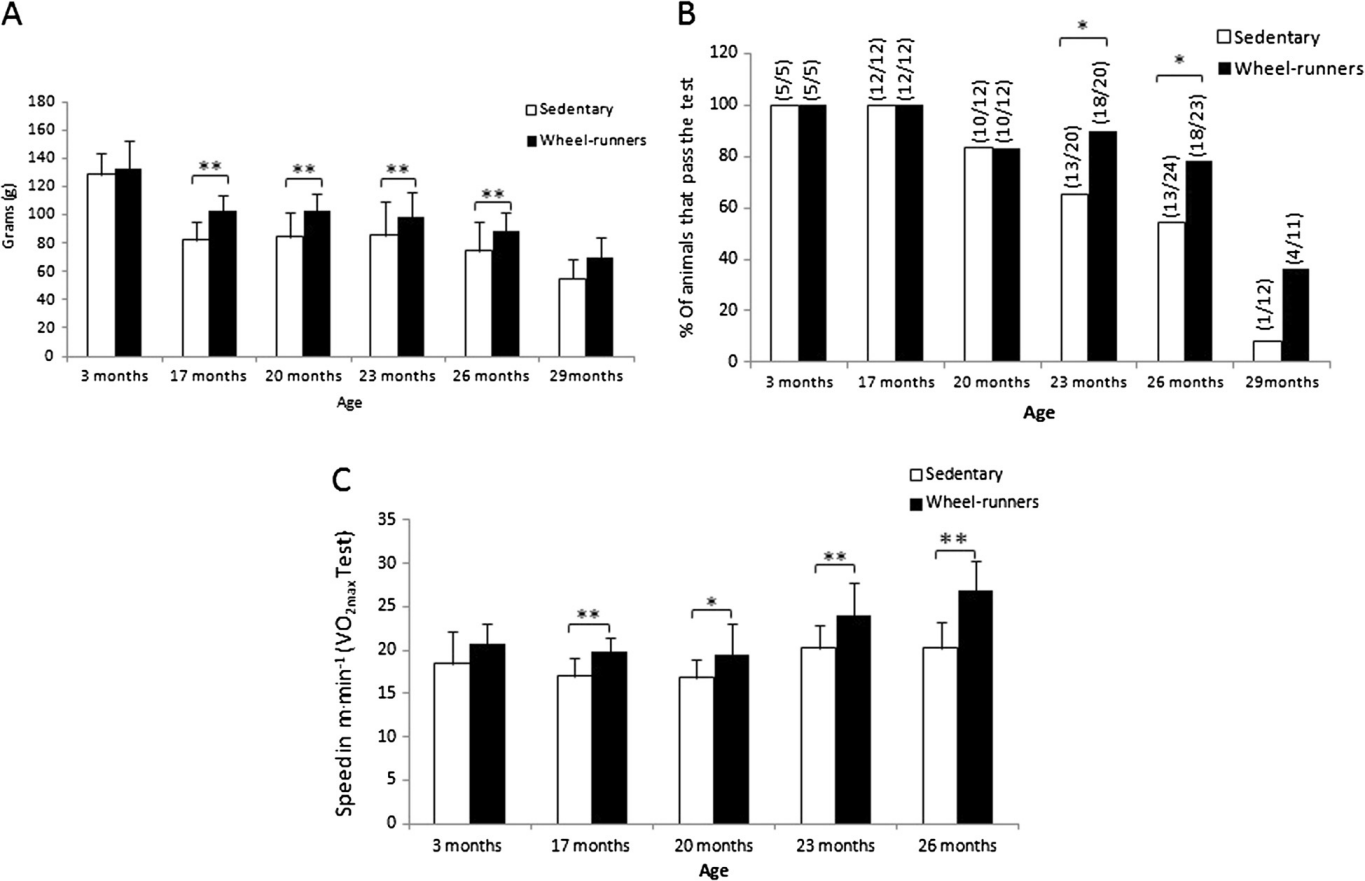
**Functional tests performed at different survival time points (3, 17, 20, 23, 26 and 29 months old) in the longevity curve. A)** shows the mouse grip strength values in grams. All the available animals were tested at the different ages. **B)** shows motor coordination. It was determined as the percentage of animals that successfully passed the tightrope test. The fraction of mice passing the test is indicated above the bars. The number of animals tested (n = 12 to 24) varied at the different ages. **C)** shows the maximal running speed achieved in a VO_2max_ test and that was considered the maximal aerobic workload capacity of the animal (n = 20). Values are shown as mean ± SD. (*) indicates *P* <0.05, (**) indicates *P* <0.01 *versus* the sedentary group at the different ages. Continuous lines show statistically significant differences between the sedentary and the wheel-running groups. VO_2max,_ exercise capacity.

Loss of grip strength is strongly associated with increasing chronological age [16] and it appears to be a powerful index of frailty. Lower grip strength is associated with incident as well as prevalent disability, suggesting that age-related loss of muscle mass and volitional muscle strength can be a cause, as well as a consequence, of physical disability [17]. We found a progressive decline in grip strength as the animals grew older in both the sedentary and the active mice. However, those mice that had free access to the running wheel had significantly higher grip strength values (*P* <0.01) than the sedentary ones at 17, 20, 23, and 26 months of age (See Figure 2, Panel A). Initial analysis for grip strength (month 3) indicated no difference between the groups.

### Motor coordination

Neuromuscular coordination declines with aging [18]. The tightrope test, which we have used in the past [19,20], is a widely used and well-validated behavioral marker of aging [21]. Neuromuscular coordination was estimated by quantifying the percentage of mice that successfully passed the tightrope test and was considered as a good marker of a decrease in physical and neuromuscular function (See Methods section for more details). Figure 2 (Panel B) shows that as age advances, the percentage of mice that did not pass the test increased in both groups, yet the spontaneous exercise group, with more passing members, had better results (*P* <0.05) than the control group in the fourth and fifth periods tested (23 and 26 months of age).

### VO_2max_ test

Large-scale epidemiological studies of subjects with and without cardiovascular disease demonstrate that low aerobic exercise capacity (VO_2max_) is a stronger predictor of mortality than other established risk factors, such as diabetes, smoking, body mass index (BMI) >30, hypertension and chronic obstructive pulmonary disease (COPD) [22-25]. There is a uniform rate of decline in VO_2max_ with age [26] and poor endurance has been considered as one of the five criteria to define frailty [7]. We found a significant difference in VO_2max_ between the runners and the sedentary animals in the test performed at the different survival time points (Figure 2, Panel C). The spontaneous wheel-running mice showed a significant increase in the maximal speed at which VO_2max_ was attained at 17, 20, 23 and 26 months of age. Initial analysis of VO_2max_ (month 3) indicated no difference between the groups.

### Mitochondrial biogenesis in skeletal muscle

**Figure 3:**
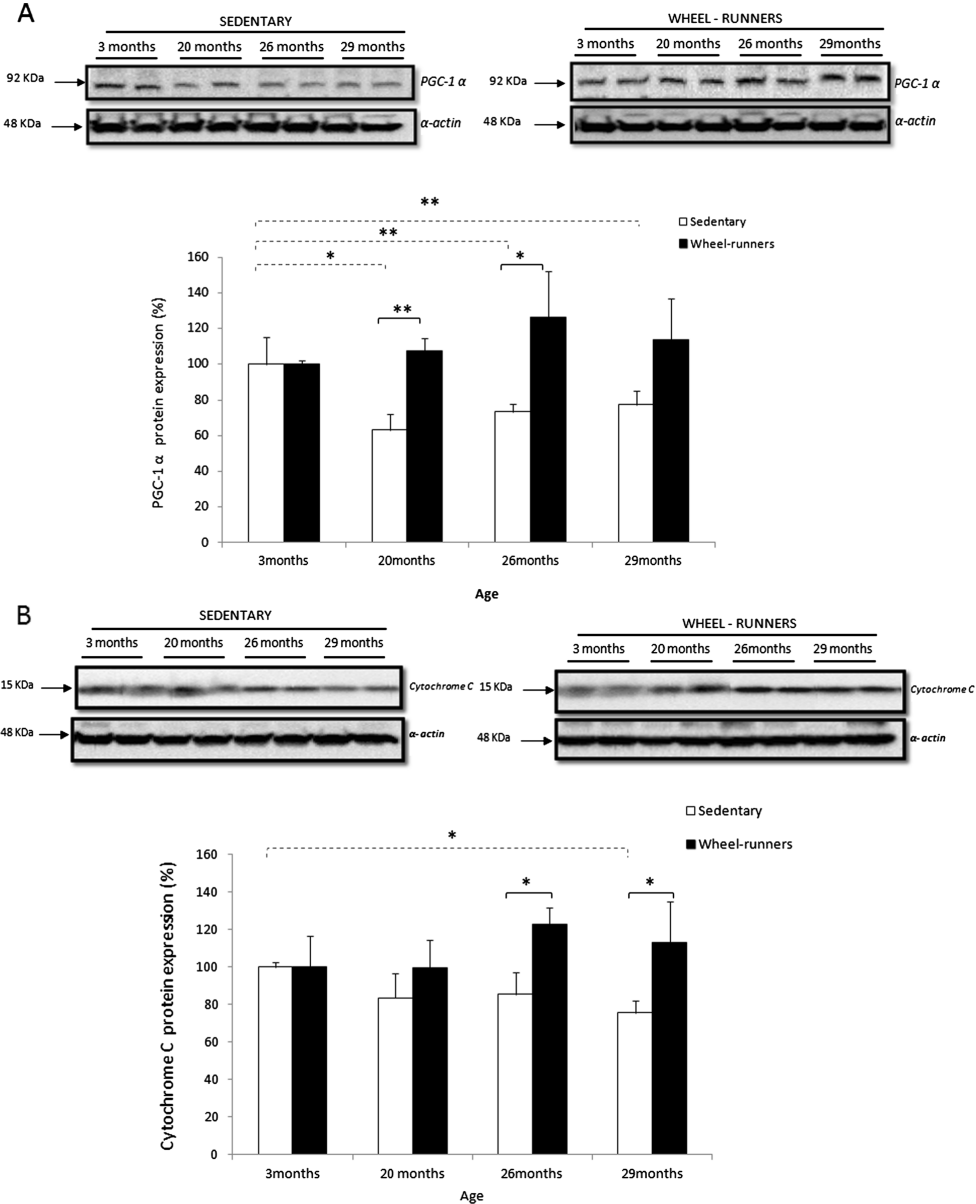
**Exercise-induced activation of the mitochondrial biogenesis pathway in mice skeletal muscle during aging.** Western blotting analysis to detect **(A)** Peroxisome Proliferator-Activated Receptor-γ Coactivator 1α (PGC-1α) and **(B)** cytochrome C at different survival time points. Representative blots are shown. For the densitometric analysis of the results, values are shown as mean (± SD). The content of α-actin, a housekeeping protein marker in skeletal muscle, was determined in all the experimental groups. (*) indicates *P* <0.05, (**) indicates *P* <0.01 *versus* the sedentary group at different ages. Values were normalized to those observed in the samples obtained from the three-month-old group, which was assigned a value of 100%. Continuous lines show statistically significant differences between the sedentary and the wheel-running groups. The discontinuous lines show statistically significant differences between the periods studied in the sedentary group.

Aging causes a decrease in mitochondrial content and activity [27-29]. Figure 3 (Panel A) shows that there was a decrease (*P* <0.01) in the protein levels of Peroxisome Proliferator-Activated Receptor-γ Coactivator 1α (PGC-1α) in skeletal muscle during aging in the sedentary animals. However, spontaneous wheel-runners maintained, or even increased, their PGC-1α levels (*P* <0.05).

Mitochondrial content was estimated measuring cytochrome C protein levels in skeletal muscle [30]. Exercise caused a significant increase (*P* <0.05) in mitochondrial content, especially in the later stages of life, that is, when mitochondria become more critical to preventing age-associated energy decay (See Figure 3, Panel B).

### Oxidative stress and antioxidant enzymes

**Figure 4:**
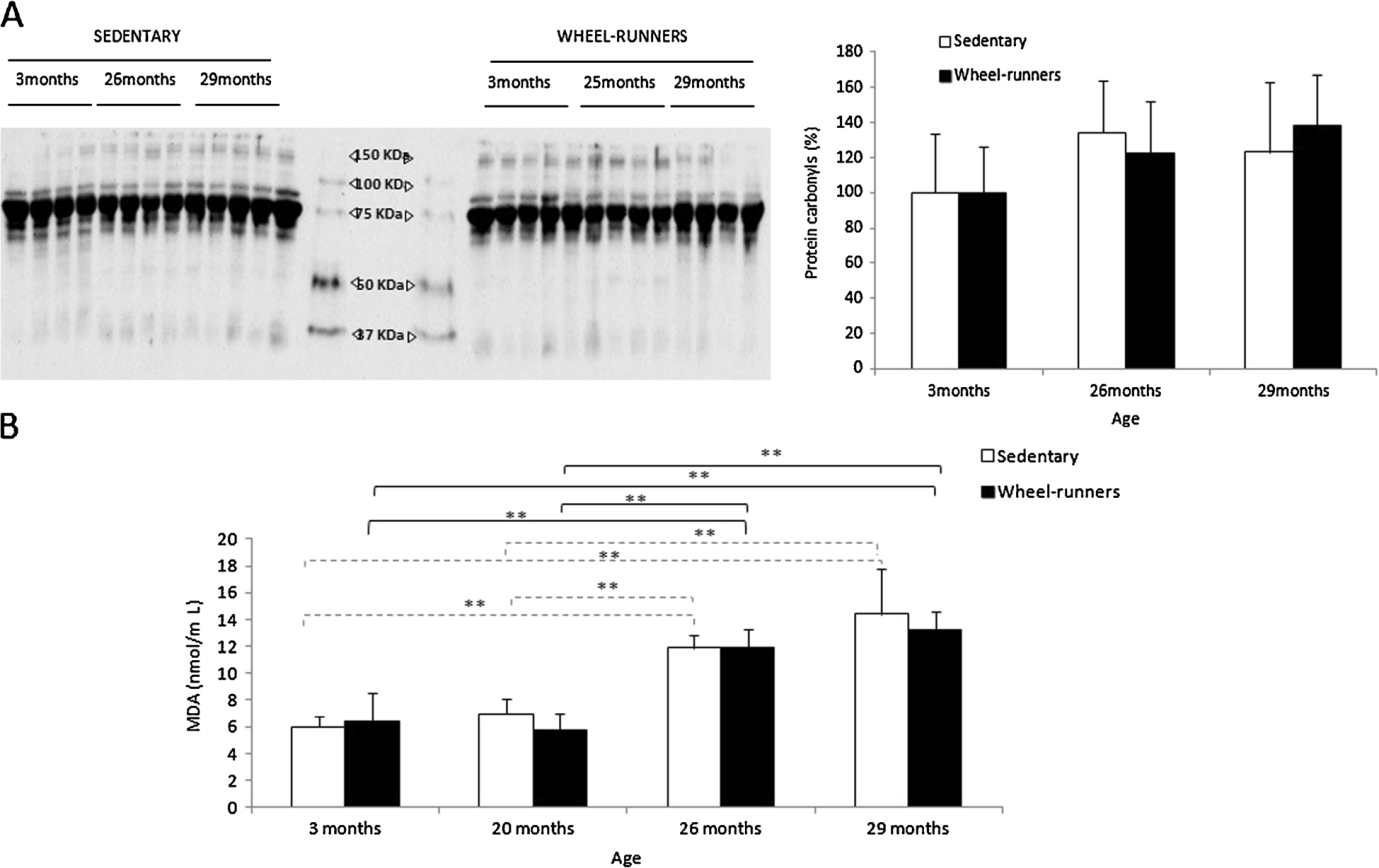
**Plasma oxidative stress biomarkers at different survival time points (3, 20, 26 and 29 months old) in the longevity curve. A)** shows a representative Western blot and the densitometric quantification of protein carbonyls in plasma. Values were normalized to those observed in the samples obtained from the three-month-old group, which was assigned a value of 100%. **B)** shows plasma lipid peroxidation determined as malondialdehyde {MDA). Values are shown as mean ± SD. (*) indicates *P* <0.05, (**) indicates *P* <0.01. Continuous lines show statistically significant differences between the wheel-running animals. Discontinuous lines show statistically significant differences between the sedentary animals.

We did not find any change in plasma protein oxidation (Figure 4, Panel A). However, we found a significant increase (*P* <0.01) in plasma lipid peroxidation (determined as malondialdehyde (MDA)) in both the sedentary and the active animals as they aged (See Figure 4, Panel B). The MDA plasma levels at 29 months of age were twice those found in the 3-month-old animals, independent of the experimental group. Thus, spontaneous wheel-running did not prevent the aging-associated increase in oxidative stress.

**Figure 5:**
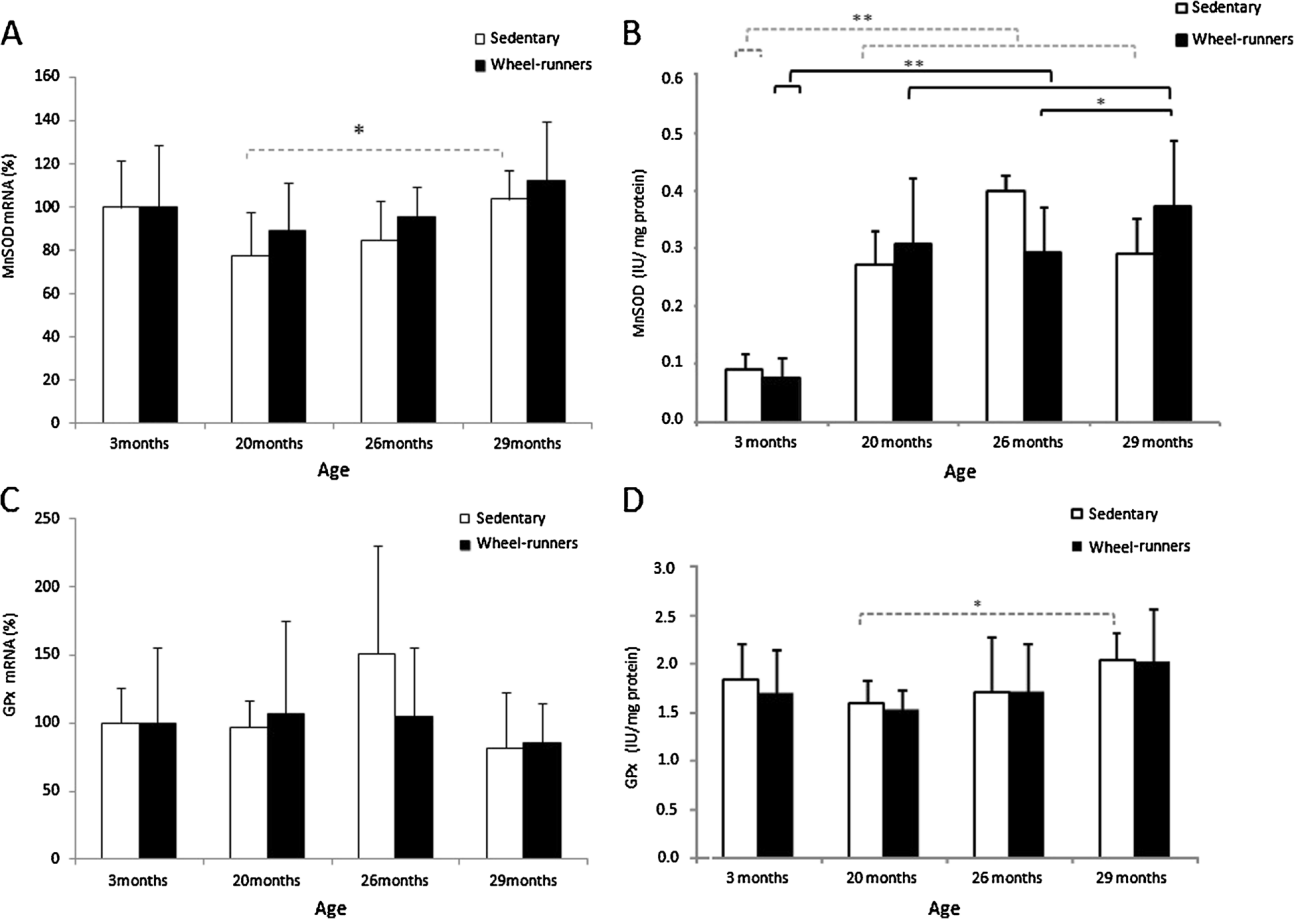
**Expression and activity of GPx and MnSOD in the liver at different survival time points (3, 20, 26 and 29 months old) in the longevity curve. A)** and **C)** show the expression of GPx and MnSOD studied by real time RT-PCR. Values were normalized to those observed in the samples obtained from the three-month-old group, which was assigned a value of 100%. **B)** and **D)** show GPx and MnSOD activity. Values are shown as mean ± SD. (*) indicates *P* <0.05, (**) indicates *P* <0.01. Continuous lines show statistically significant differences between the wheel-running animals. Discontinuous lines show statistically significant differences between the sedentary animals. GPx, glutathione peroxidase; MnSOD, manganese superoxide dismutase.

We also determined the expression and the activity of the antioxidant enzymes MnSOD and GPx. We found no major changes in the mRNA levels of the enzymes (See Figure 5, Panels A and C). However, their activity was elevated in the livers of the animals as they grew older (See Figure 5, Panels B and D).

### Cortical BDNF

Protein levels of BDNF were quantified in the cortex of the animals by ELISA. We found a significant increase in the neurotrophin in the active animals. Thus, spontaneous wheel-running significantly prevented the age-associated fall in BDNF in mice 20- and 26-months old.

## Discussion

Most of the interventions devised to understand the mechanisms of aging have been focused on survival [4]. Recently, however, emphasis has been placed on preventing disability (healthspan) and its predictors (frailty) rather that on merely increasing longevity (lifespan).

We have recently proposed that ‘exercise acts as a drug’ [31]. The beneficial effects of regular exercise for the promotion of health and cure of diseases have been clearly established in humans [11,32-34] as well as in rodents [12,35,36]. We did not find any effect of lifelong spontaneous exercise on longevity (See Figure 1). Exercise has been unequivocally associated with a slowing of age-specific mortality increases in rats and with an increased median lifespan [36]. However, the results in mice are not that clear. In 1984, it was shown that no significant differences in lifespan were found in mice that had free access to running-wheels during senescence and/or maturity [37]. In 2004, it was shown that moderate exercise, provided by weekly treadmill training (10, 15, and 20 cm.s^-1^ for 5 minutes each, every 7 days) starting at 28 wks of age, increased survival in CD-1 mice (median lifespan was increased by 19% and maximal lifespan was increased by 15% to 21% in males) [12]. On the contrary, we did not find a significant difference on average or maximal lifespan in the wheel-running group, although a statistical trend (*P* = 0.09) was found. These different findings may be explained by the different experimental conditions. Essentially, we housed our mice in a clean animal house and we did not force the animals to exercise. Moreover, we used C57BL/6J mice (which do not gain weight with age) whereas CD-1 mice and Wistar rats do. We believe that C57BL/6J mice are more suitable for studying exercise and aging because the effects are free from the confounding factor of the exercise-induced loss of weight that may occur in animals that become obese with aging. In 2003, Bronikovski and co-workers, using lines of outbred genetically heterogeneous mice selectively bred for high spontaneous exercise, found that exercise increased median (17%), but not maximum, lifespan in rodents [38]. In this case, differences not only in the strain but in the number of kilometers run per day between their animals and ours could explain these differences. These authors reported an average of 4.8 km.day^-1^ in 20-month-old animals while in our experiment the animals ran 1 km.day^-1^ at the same age.

We chose the C57BL/6J mouse as the strain for our model for three main reasons. First, this strain has been shown to perform well in spontaneous exercise studies [39]. Second, by using an inbred strain, we intended that all animals would begin with a nearly identical genotype (99.9% genetically identical), thereby increasing the probability that any phenotypic variation would be strictly due to environmental influences. Finally, (unlike rats or humans) C57BL/6J mice do not gain weight as they age and exercise does not cause a weight loss in these animals. Thus, because the level of spontaneous exercise was the only known variable between groups, any differences would be related to spontaneous exercise.

To support our results we determined oxidative stress parameters and the activity of the antioxidant enzymes in our study. The free radical theory of aging is one of the most prominent theories to explain aging. This theory, although recently questioned [40], has been tested in various laboratories and there are many published papers in its support [41]. As expected, we found a significant increase in plasma lipid peroxidation in the old animals, in both the active and inactive groups (See Figure 4). We also wanted to study the antioxidant status by measuring the expression and activity of the antioxidant enzymes MnSOD and GPx. Genes that encode antioxidant enzymes are considered longevity genes because their over-expression may modulate lifespan [42]. We found a significant increase in antioxidant enzyme activities (especially in MnSOD) as the animals grew older, independent of their physical activity (See Figure 5, Panels B and D). It has been well documented that several tissues increase their antioxidant enzyme activities as they age [43,44]. However, we did not find any significant changes in the relative abundance of mRNA for the enzymes (See Figure 5, Panels A and C). Our results suggest that age-related increases in MnSOD and GPx activity were not caused by enhanced gene expression but by a posttranslational modification (activation) of the enzyme molecules in aged liver [45].

The negative results in terms of lifespan led us to focus our study on healthspan. Ignatz Nascher, who coined the term ‘geriatrics’ and who founded this clinical field in the US, described the concept of healthspan (without using the term) as a goal of being productive and happy for an individual’s entire lifespan, rather than seeking longevity despite severely hindering impairments of body and mind [2,46].

Frailty is a geriatric syndrome with a tremendous impact on the older individual, their family, and society as a whole. The components of frailty are a mixture of physiological, psychological, social and environmental factors (for example, sarcopenia, functional impairment, cognitive impairment, and depression). Physical exercise may affect all these factors. Thus, we aimed to determine whether life-long spontaneous exercise was a good strategy to prevent frailty in a mouse model. Our major problem was the lack of a test for frailty in experimental animals. Although the clinical interest in frailty has grown in recent years [47], research in experimental animal models of frailty is very rare. The most commonly used test for frailty is that of Linda Fried and co-workers [7]. They concluded that frailty is a combination of five components: unintentional weight loss, exhaustion, weakness, slow running speed and a decrease in physical activity. Based on this work, three functional tests were performed on our mice at six different survival time points (See Figure 2). We found that as the animals grew older, they showed poorer results in the tests that determined healthspan: grip strength (Panel A), motor coordination (Panel B) and aerobic exercise capacity (Panel C). Mice that had free access to running wheels performed significantly better than the sedentary animals in all the tests.

**Figure 6:**
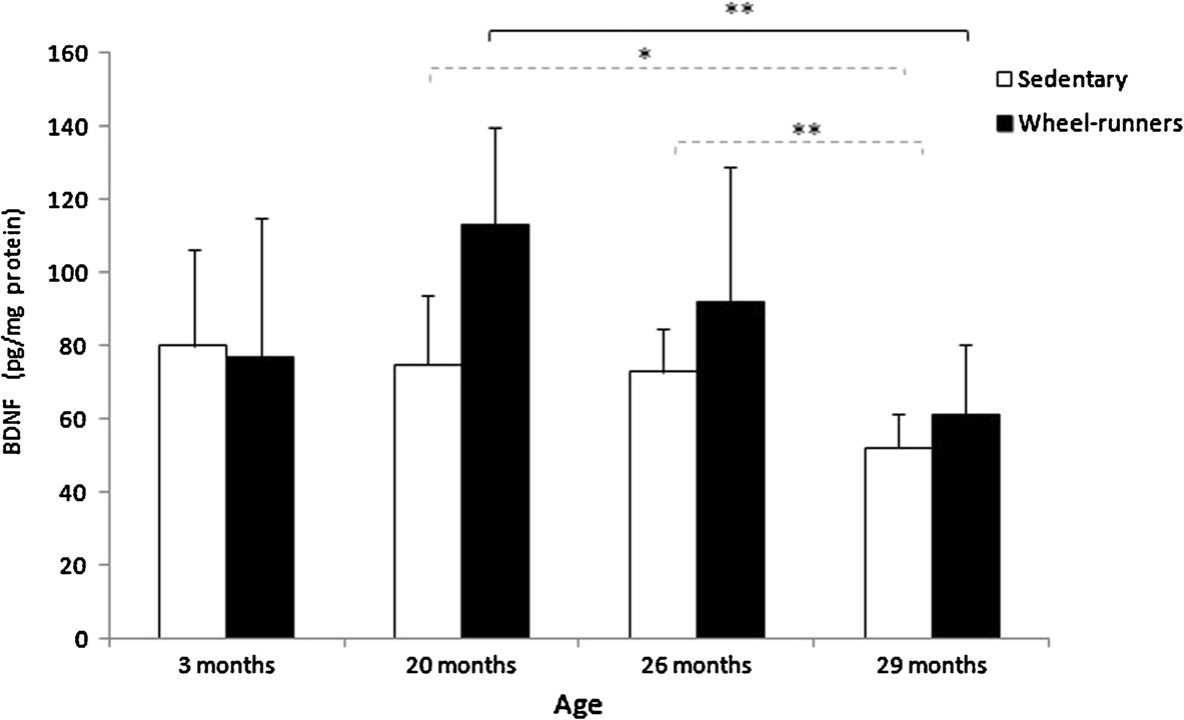
**Cortex BDNF levels, determined by ELISA, in sedentary and wheel-running mice at different survival time points (3, 20, 26 and 29 months old) in the longevity curve.** Values are shown as mean ± SD. (*) indicates *P* <0.05, (**) indicates *P* <0.01. Continuous lines show statistically significant differences between the wheel-running animals. Discontinuous lines show statistically significant differences between the sedentary animals. BDNF, brain-derived neurotrophic factor.

The beneficial effects of exercise on cognitive [48] and skeletal muscle function may be mediated by two adaptations: increased expression of neurotrophic factors in some brain areas [49] and the induction of mitochondrial biogenesis in skeletal muscle [50]. BDNF is a neurotrophic factor that may play an important role in old-age survival, because of its role in preventing neuronal death during stress [51] and in synaptic plasticity [52]. Plasma concentrations of BDNF are significantly higher in non-frail than in pre-frail women [52]. We observed that BDNF levels fall with age in sedentary animals but, remarkably, they increase with exercise in old animals and only fall in very old ones (that is, 29 months old) (See Figure 6).

A functional muscle that has not lost the capacity to synthesize healthy mitochondria is an important contributor in the prevention of frailty [47,53]. Thus, we determined two relevant proteins involved in mitochondrial biogenesis in skeletal muscle, PGC-1α and cytochrome C. We recently showed that aging causes a lack of response of PGC-1α to various stimuli [29]. We hypothesized that lifelong spontaneous exercise could prevent the lack of reactivity of PGC-1α in the aging muscle and this turned out to be the case (See Figure 3, Panel A). We estimated the mitochondrial content by measuring cytochrome C protein levels [30]. Spontaneous exercise resulted in a significantly higher level of this protein at 26 and 29 months of age when compared with the sedentary animals (See Figure 3, Panel B). PGC-1α protects against skeletal muscle atrophy [54] and it is required for training-induced prevention of the age-associated decline in mitochondria [55]. Moreover, it has been recently shown that increased muscle PGC-1α expression protects from sarcopenia and metabolic disease during aging [56]. Our results confirm that life-long spontaneous exercise contributes to the maintenance of the mitochondrial content in skeletal muscle during aging.

## Conclusion

Lifelong spontaneous exercise does not prolong lifespan but improves healthspan in mice. Exercise is an intervention that enhances function and delays frailty in experimental animals. These results stress the importance of this intervention to prevent human frailty and dependency.

## Methods

### Experimental animals

Adult male C57BL/6J mice, three-months old, were randomly assigned to one of two groups: sedentary control (n = 72) or spontaneous exercise (n = 72). The animals were housed in individual cages. The mice in the exercise group had 24-hour access to a 11.5 cm diameter running wheel connected to an electronic wheel-revolution counter located at the top of the cage. The sedentary mice were free to move around their cage but did not have access to a running wheel. We chose to wait until three months of age to allow our animals access to exercise wheels, as this is the age at which mice reach musculoskeletal maturity, and we estimated that our animals would achieve maximal performance if they were exposed to running wheels at this age [57].

The average temperature in the animal house was 23 ± 1°C, relative humidity was 60%, and 12 hour day/night cycles were maintained. The mice were checked daily. Water and food were available *ad libitum*. Food consumption was determined weekly by subtracting the amount of food remaining from the amount offered. No differences between the sedentary and the wheel-runners were found (data not shown). The cage bottoms were cleaned once every fortnight and wheels once every four weeks.

The values obtained in the longevity curve were registered for as long as the experiment lasted, sacrificing four mice from each group at specific survival points: 3, 20, 26 and 29 months of age, to obtain data for subsequent analysis. Liver, skeletal muscle and brain were immediately dissected and stored at −80°C for further analysis.

The experimental protocol was approved by the Committee of Ethics in Research of the Faculty of Medicine, University of Valencia.

### Wheel running activity

The activity of the mice on the running wheels was monitored by a magnetic switch affixed to each wheel, which recorded the number of revolutions completed. Physical activity was recorded continuously and added up each week for analysis. Free open-field locomotor activity of mice within cages was not measured.

### Motor coordination test (tight-rope test)

The tight-rope test was based on the method previously described by Miquel [21] and extensively used by our team [19]. Mice were placed in the middle of a 60 cm long and 1.5 cm wide rope. The test results were considered successful if the mouse reached either the end of the rope or if it was able to stay on it for 60 seconds. All the animals had five chances to complete the test. We determined the percentage of mice that succeeded in passing the test. This test was performed at six different time points in the longevity curve (3, 17, 20, 23, 26 and 29 months of age).

### Incremental treadmill test (VO_2max_ test)

The animals were submitted to a graded intensity treadmill test (Model 1050 LS Exer3/6; Columbus Instruments, Columbus, OH, USA) to determine their endurance and ‘slowness’ along the longevity curve. We followed a modification of the protocol of Davidson and co-workers [58]. After the warm-up, the treadmill band velocity was increased until the animals were unable to run further. The initial bout of 6 minutes at 6 m..min^-1^ was followed by consecutive 2 m.min^-1^ increments every two minutes. Exhaustion was defined as the third time a mouse could no longer keep pace with the speed of the treadmill and remained on the shock grid for two seconds rather than running. Exercise motivation was provided for all rodents by means of an electronic shock grid at the treadmill rear. However, the electric shock was used sparingly during the test. The maximal running speed was considered the maximal aerobic workload capacity of the animal (22). This test was performed at five different time points in the longevity curve (3, 17, 20, 23 and 26 months of age). We could not perform the test when the animals were 29 months old because they ran less than 0.1 km daily.

### Grip strength test

A grip strength meter (Panlab, Harvard Apparatus. Barcelone. Spain) was employed in assessing neuromuscular function by sensing the peak amount of force that the mice applied in grasping specially designed pull bar assemblies. Metering was performed with precision force gauges in such a manner as to retain the peak force applied on a digital display. Mice were randomly chosen to grasp the pull-bar with their forelimb for a few seconds. The animals were then drawn along a straight line leading away from the sensor. The animals released at some point and the maximum force attained was stored on the display. Peak force was automatically registered in grams-force by the apparatus. Data were recorded, and four additional trials were immediately given [59]. This test was performed at six different time points in the longevity curve (3, 17, 20, 23, 26 and 29 months of age).

### SDS-PAGE and western blotting

Aliquots of muscle lysate were separated by SDS-PAGE. Proteins were then transferred to nitrocellulose membranes, which were incubated overnight at 4°C with appropriate primary antibodies: anti-PGC-1α (1:1000, Cayman. Ann Arbor. Michigan. USA), anti-cytochrome C (1:1000, Santa Cruz Biotechnology Inc. Dallas. Texas. USA), and anti-α-actin (1:700, Sigma Aldrich. St. Louis. Missouri. USA). Thereafter, membranes were incubated with a secondary antibody for one hour at room temperature. Specific proteins were visualized by using the enhanced chemiluminescence procedure, as specified by the manufacturer (Amersham Biosciences, Piscataway, NJ, USA). Autoradiographic signals were assessed by using a scanning densitometer (BioRad, Hercules, CA, USA). The densitometry analysis was carried out immediately before saturation of the immunosignal. Data were represented as arbitrary units of immunostaining. To check for differences in loading and transfer efficiency across membranes, an antibody directed against α-actin was used to hybridize with all the membranes previously incubated with the respective antibodies.

### Determination of plasma MDA and protein carbonyls

MDA was determined in plasma by an HPLC method as described in [60]. Oxidative modification of total proteins was assessed by immunoblot detection of protein carbonyl groups using the ‘OxyBlot’ protein oxidation kit (Millipore. Madrid. Spain) following the manufacturer’s instructions. Approximately 20 μg of total protein was loaded onto paired gels and electrophoretically separated (see previous section). Antibody anti-dinitrophenylhydrazone was purchased from Intergen. The procedure to quantify total protein carbonyls with the OxyBlot kit used densitometry of the oxyblot and of the Ponceau staining, followed by finding the ratio between the total density in the oxyblot and the total density in the Ponceau [19].

### RNA isolation, reverse transcription and PCR

Total RNA was extracted from liver tissue with Trizol™ (Invitrogen. Madrid. Spain) according to the manufacturer’s protocol. The purity of the samples was assessed by determining the 260 nm/280nm ratio, which was always above 1.9, and total RNA was quantified from the absorbance at 260 nm. We synthesized cDNA from 1 μg of RNA using random hexamer primers and the High Capacity cDNA Reverse Transcription Kit (Applied Biosystems, Madrid, Spain). Reverse transcription conditions comprised an initial incubation step at 25°C for 10 minutes to allow random hexamers to anneal, followed by cDNA synthesis at 37°C for 120 minutes and the final inactivation step for 5 minutes at 95°C. Real-time PCR was performed with an ABI 7900 sequence-detection system (Applied Biosystems). Primers for amplifying specific fragments of the genes were obtained from Thermo Fisher Scientific GmbH (Ulm, Germany). Real-time PCR was performed in duplicate in a total reaction volume of 20 μL using Maxima™ SYBR green/ROX qPCR Master Mix (Fermentas, Madrid, Spain). The thermal cycling protocol was as follows: initial denaturation for 10 minutes at 95°C followed by 40 cycles of 10 seconds at 95°C, 10 seconds at 62°C, and 10 seconds at 72°C. The fluorescence signal was measured at the end of each extension step at 72°C. At the end of each reaction, a melting curve analysis was performed to confirm that only the specific products were amplified. The threshold cycle (Ct) was converted to a relative gene expression by the use of a standard curve. For each sample, the expression of the target gene mRNA was normalized with the GAPDH mRNA content. The specific primers used for GPx were: 5’-GAC ATC AGG AGA ATG GCA AG-3’ (forward) and 5’- CAT CAC CAA GCC AAT ACC AC-3’ (reverse); for MnSOD they were: 5’-CGT GCT CCC ACA CAT CAA TG-3’ (forward) and 5’-TGA ACG TCA CCG AGG AGA AG-3’ (reverse); and for the housekeeping gene GAPDH they were: 5’- CCT GGA GAA ACC TGC CAA GTA TG-3’ (forward) and 5’-GGT CCT CAG TGT AGC CCA AGA TG-3’ (reverse).

### Enzyme activities

GPx activity was measured as described by Flohe *et al*. [61]. SOD was determined following the instructions of the ‘Superoxide Dismutase Assay Kit’ (Caymen Chemical).

### ELISA analysis

Protein levels of BDNF were quantified in the cortex by ELISA (CYT306 Millipore, Bedford, MA, USA), following the manufacturer’s instructions. The samples were measured at 450 nm using a plate reader (iEMS Reader MF; Labsystems, Vantaa, Finland).

### Data analysis

Mean values and standard deviation were considered for descriptive statistics. To estimate lifespan differences between groups, a Kaplan-Meier curve was performed. Differences in maximal running time and speed, grip strength test and motor coordination were tested using Fisher’s exact test for each age group and parameter. To determine the effect of spontaneous exercise on BDNF, PGC-1α and cytochome C protein expression in skeletal muscle we performed a two-tailed Student’s t-test for unpaired samples. Differences were considered significant at *P* <0.05. Statistical calculations were performed using SPSS (version Pasw Statistics 17.0) software.

## Abbreviations

BDNF: Brain-derived neurotrophic factor; ELISA: Enzyme-linked immunosorbent assay; GPx: Glutathione peroxidase; HPLC: High performance liquid chromatography; MDA: Malondialdehyde; Mn-SOD: Manganese superoxide dismutase; PCR: Polymerase chain reaction; VO2max: exercise capacity.

## Competing interests

The authors declare that they have no competing interests.

## Authors’ contributions

RG-V and GO-G performed the experimental work; MCG-C, FJG-G and LR-M analyzed data and assisted in editing and writing the paper; DA, and NI supervised all the animal work and designed research; and J V wrote the paper and directed the project. All authors read and approved the final manuscript.
